# Expression of tumor suppressors miR-195 and let-7a as potential biomarkers of invasive breast cancer

**DOI:** 10.6061/clinics/2018/e184

**Published:** 2018-06-29

**Authors:** Marcia M. Marques, Adriane F. Evangelista, Taciane Macedo, René Aloisio da Costa Vieira, Cristovam Scapulatempo-Neto, Rui M. Reis, André L. Carvalho, Ismael Dale Cotrim Guerreiro da Silva

**Affiliations:** I Centro de Pesquisa em Oncologia Molecular (CPOM), Hospital de Cancer de Barretos, Barretos, SP, BR; II Faculdade de Ciencias da Saude de Barretos Dr Paulo Prata (FACISB), Barretos, SP, BR; III Departamento de Mastologia e Reconstrucao Mamaria, Hospital de Cancer de Barretos, Barretos, SP, BR; IV Departamento de Patologia, Hospital de Cancer de Barretos, Barretos, SP, BR; V Instituto de Pesquisa de Ciencias da Vida e Saude, Universidade de Minho, Braga, Portugal; VI Laboratorio Associado ICVS/3B’s, Braga, Portugal; VII Departamento de Ginecologia, Universidade Federal de Sao Paulo, Sao Paulo, SP, BR

**Keywords:** Breast Cancer, Biomarkers, Tumor Suppressors, Early Detection, microRNAs

## Abstract

**OBJECTIVE:**

MicroRNAs (miRNAs) are small non-coding RNAs that regulate gene expression at the posttranscriptional level. Some miRNAs, including let-7a and miR-195, have been described as tumor suppressors. However, the roles of these microRNAs in breast cancer progression remain controversial. The aim of this study is to evaluate miR-195 and let-7a expression as potential biomarkers of invasive breast cancer.

**METHODS:**

In the present study, 200 individuals were separated into three groups: (i) 72 women constituting the control group who were selected according to rigorous and well-established criteria; (ii) 56 patients with benign breast tumors; and (iii) 72 patients with malignant breast cancers of different clinical stages. The miR-195 and let-7a expression levels in serum were evaluated by real-time PCR. The results were assessed alone and in combination, and the analysis included an estimation of sensitivity and specificity in ROC curves.

**RESULTS:**

Compared with the benign and control groups, both microRNAs were downregulated in the malignant breast cancer patient group. Compared with the malignant group, the combination of both biomarkers in the control and benign groups showed good sensitivity and specificity in the serum with AUCs of 0.75 and 0.72, respectively. The biomarker combination for the control group *versus* the malignant group exhibited a better sensitivity and specificity than for the benign group *versus* the malignant group.

**CONCLUSION:**

These findings support the evidence that the analysis of miR-195 and let-7a can be used as a non-invasive biomarker for breast cancer detection.

## INTRODUCTION

MicroRNAs (miRNAs) are small non-coding RNAs that are approximately 22 nucleotides in length, and these RNAs regulate gene expression at the posttranscriptional level 1. These molecules are conserved across species and are deregulated in several diseases, including cancer 2–4. Evidence has shown that miRNAs are located on fragile sites of the genome and/or cancer-associated regions and act either as oncogenes or tumor suppressors 5.

The tumor suppressor miRNAs of the let-7 family regulate important oncogenes, such as c-*MYC*, H-*RAS*, and *HMGA2*, and several cell cycle regulators 6–8. Let-7a is the most conserved of the let-7 family members, exhibiting high sequence similarity between many organisms from *Caenorhabditis elegans* to humans 7. Let-7a is frequently downregulated in several types of cancers 8. Let-7a is regulated by p53 and its homologues, demonstrating the tumor suppressive properties of these regulators 9. In addition, let-7a is involved in glucose metabolism 10 and angiogenic mechanisms 11.

In breast cancer cells, let-7 processing is affected by *LIN28,* which binds to the terminal loops of let-7 precursors, leading to the inhibition of its processing and the induction of uridylation 12. A recent study reported that *LIN28* decreased Myc expression after MAPK signaling inhibition, which consequently led to let-7 induction and the suppression of breast cancer bone metastasis in animal models 13. Raf kinase inhibitory protein (RKIP) can regulate *LIN28* and, consequently, let-7 in breast cancer 13. In addition, other forms of let-7 regulation have been shown in breast cancer. For example, let-7 regulation can occur via miRNA regulation by other miRNAs, such as the interaction with miR-107, which downregulates let-7 during tumor progression 14.

Another important miRNA tumor suppressor, miR-195, is a member of the miR-15/16/195/424/497 family, which is characterized by the same 3′ untranslated region (3′UTR) binding seed sequence and similar bases AGCAGC near the 5′ ends of mature miRNAs 15. The tumor suppressor function of miR-195 has been associated with the downstream effectors of NF-kB, especially IKKa and TABP3, linking inflammation to tumorigenesis 16. miR-195 downregulation has been observed in several types of cancers and may be associated with the copy number loss of a segment of chromosome 17p13.1, as observed in ovarian serous carcinoma 17. Moreover, epigenetic alterations, namely, promoter CpG methylation, have also been associated with miR195 downregulation in gastric cancer, with consequent deregulation of its direct target *CDK6* 18.

In hepatocellular carcinoma, miR-195 induces G1 arrest by affecting cell cycle regulators 19 and blocking the G(1)/S transition by repressing Rb-E2F signaling through the targeting of *CCND1*, *CDK6*, and *E2F3* 20. This microRNA has been described as a downregulator of *WEE1*, the major gate keeper at the G2 cell cycle checkpoint, and affects the migration and invasion of melanoma cells 21. In addition, another miR-195 target, *TARBP2*, affects cell proliferation and apoptosis and seems to discriminate between adenomas and carcinomas 22. In breast cancer, miR-195 downregulation is affected by the methylation status of the CpG island 23. Using luciferase assays, Li et al. 23 showed that the *RAF-1* and *CCND1* transcripts were direct targets of miR-195 in breast cancer. The authors of this study also performed a Western blot analysis of both targets in several breast cancer cell lines to validate the regulation of these targets after miR-195 transfection.

A recent study by Heneghan et al. 24 demonstrated that miR-195 expression could differentiate breast cancer from other cancers. Indeed, the upregulation of miR-195 and let-7a showed a significant correlation with clinicopathological variables, such as nodal status and estrogen receptor status 25. Moreover, the downregulation of miR-195 expression was observed in an athymic mouse model of breast cancer 26.

The possibility of miRNA detection in cell-free forms, such as exosomes, together with specific properties, such as tissue specificity and stability, indicates that miRNAs are important biomarkers 27. In this study, we aimed to assess whether the miR-195 and let-7a expression levels in the serum samples of breast cancer patients could be used as biomarkers of breast carcinogenesis. The selection of these microRNAs is based on the existing contradictory results in the literature.

## MATERIALS AND METHODS

### Patient selection

The current prospective controlled exploratory study was approved by the local Ethical Research Committee (protocol #272/2009). The patients were selected according to their clinical features and pathological biopsy data. They were categorized into three groups (Table 1). Group I was considered a control group and included women between 40 and 69 years of age, with Gail scores <1.66 and normal radiological examinations 28. Group II, the benign group, constituted women with mammary biopsies and radiological alterations (BI-RADS IV) or clinical pathological discordance in which the pathological findings showed the absence of cancer. In this group, lesions were considered benign and low risk, with the exclusion of atypical and higher-risk alterations. Finally, group III comprised randomly selected patients with invasive ductal carcinoma, and the inclusion criteria were associated with the patients’ consent with the study and the RNA quality of the samples. The AJCC 7^th^ classification (TNM) was used for clinical staging 29.

### Blood sample collection

Blood was collected in BD Vacutainer SST II Advance (367955) collection tubes, and serum separation was performed within 2 hours of collection. Samples were then stored at -80°C until use. Venous blood samples were collected from patients prior to any surgery/biopsy or chemotherapy or radiotherapy treatment.

In parallel, pathological data were analyzed from FFPE material with immunohistochemistry markers. Estrogen and progesterone receptors were considered positive after detection of nuclear expression ≥1%, regardless of the staining intensity. Her2 was considered positive with a 3+ score if 30% of the cells were stained. A percentage of 10-30 was considered a 2+ score, by FISH or DISH immunofluorescence. Ki67 was also measured; however, due to the low number of cases, the luminal A and B-Her2-negative molecular subtypes were grouped. Molecular classification by immunohistochemistry was performed according to Goldhirsch et al. 30.

### RNA isolation and real-time PCR (qRT-PCR) assay

The RNA isolation protocol using MiRNeasy (Qiagen, Hilden, Germany) was used for serum samples. Quantification was performed using a nanodrop ND-1000 spectrophotometer (NanoDrop Products, Wilmington, DE, USA).

For real-time PCR, 10 ng of total RNA was used to perform reverse transcriptase reactions using Taqman miRNA Assays (Life Technologies, Inc., Carlsbad, CA, USA), according to the manufacturer’s instructions. All real-time PCR reactions were run as technical triplicates in a 7900 HT Fast Real-time PCR System (Applied Biosystems USA, Foster City, CA, USA).

### Data analysis

**Figure 1:**
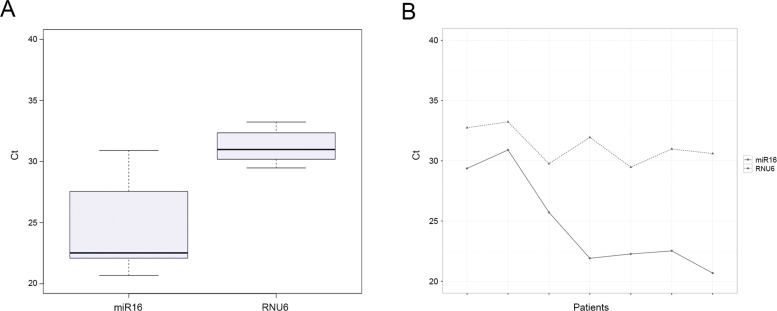
Relative quantification values of miR-16 and U6 used to normalize the control and breast cancer samples.

The analysis procedures were performed in the R environment (version 3.1.1) 31. The normalization step was performed according to the 2^-ΔΔCt^ method 32. Cycle threshold (Ct) values from the selected miRNA targets were subtracted from the Ct values of endogenous small non-coding RNA control RNU6 (Figure 1) (Control miRNA Assay, Applied Biosystems). A subsequent ΔΔCt value was calculated for the malignant tumors using the lower expression values of the control or benign ΔCt values. Expression values are represented as the ΔΔCt value on a log2 scale. Statistical analysis was performed using one-way ANOVA with a significance level of *p*≤0.05. To assess the sensitivity and specificity of each biomarker, ROC curve analysis was used. Analysis of the combination of biomarkers was performed using a general logistic model (glm). ROC analysis was performed using the ROCR package 33.

## RESULTS

### Clinical data

The features of each group are summarized in Tables 1 and 2. Group I comprised 72 healthy women, with a median age of 51.4 years (40-68) and Gail score of 1.16 (0.93-1.60). No malignancy focus was observed in this group, and the BI-RADS I, II, and III frequencies in this group were 47.2%, 48.6%, and 4.2%, respectively.

The benign group, Group II, comprised 56 women, with a median age of 49.8 years (27-79) and Gail score of 1.9 (0.93 to 6.91). All lesions in this group presented the radiological characteristics of BI-RADS IV, which was determined by mammography, mammary ultrasonography, and magnetic resonance for 44.6%, 53.6%, and 1.8% of the patients, respectively. The major pathological findings were typical ductal hyperplasia (n=20), sclerosing adenosis (n=11), fibroadenoma (n=11), simple adenosis (n=3), benign phyllodes tumor (n=2), papilloma (n=2), and apocrine metaplasia (n=2). These categories represented 91.1% of the findings.

Group III, the malignant group, comprised 72 breast cancer patients with a median age of 54 years (28-81) and a median tumor size of 2.6 cm (1-15 cm). Only 8.3% of the patients in group III had a diagnosis of metastasis. Only one patient presented with lobular carcinoma, which was observed after histological revision, and the patient was not excluded. The patient presented levels of expression that were similar to those of the other patients in the group. The main characteristics of group III were TNM II and III (72.2%), TNM-T T1/T2 (68.0%), TNM-N N0/N1 (73.6%), and luminal A/B Her2-negative, according to immunohistochemical analysis.

### Data analysis

**Figure 2:**
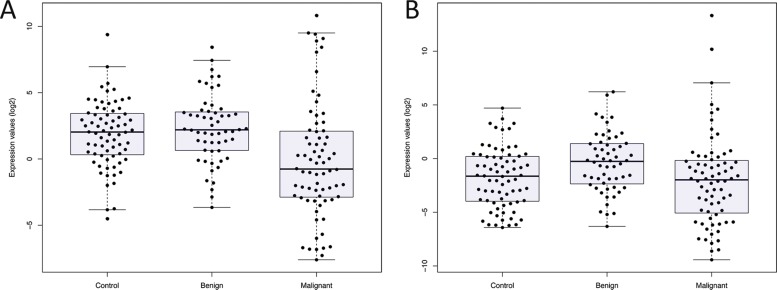
Relative quantification values are represented as log2 of the ΔΔCt value, which was calculated using RNU6 as a housekeeping gene and was adjusted with the lower expression values of the control or benign ΔCt values for the malignant tumors. (A) let-7a and (B) miR-195 expression in the sera of the control, benign, and malignant breast cancer groups.

The expression levels of miRNAs, namely, miR-195 and let7a, were assessed by qRT-PCR, and a comparison between the serum expression levels was performed for the control, benign, and malignant groups (Figure 2). We observed downregulation in both let-7a (Figure 2-A) and miR-195 (Figure 2-B) in the malignant group compared with the control and benign groups. One-way ANOVA of these data showed *p-*values of 0.0004 for let-7a and 0.006 for miR-195.

**Figure 3:**
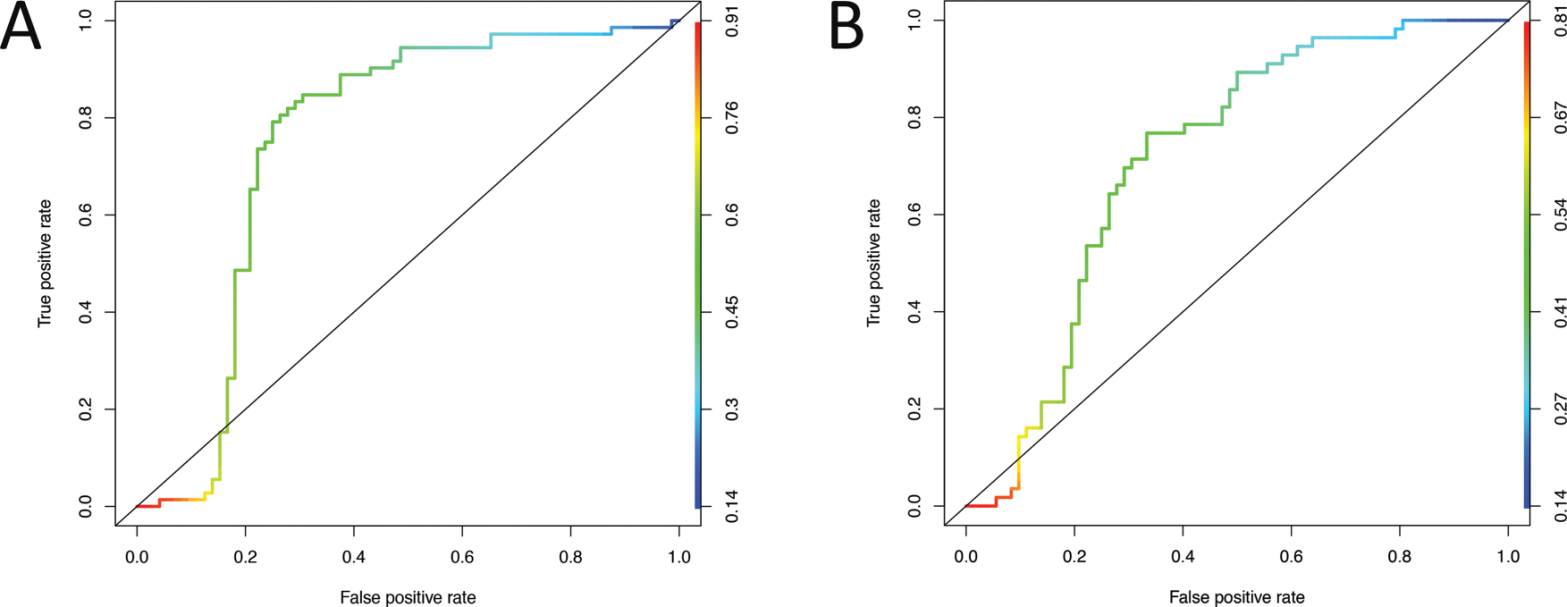
Receiver operating characteristic (ROC) curves after combining let-7a miR-195 expression values using a general logistic model. (A) ROC curve of the control group *versus* the malignant group; (B) ROC curve of the benign *versus* malignant groups.

The combination of these biomarkers showed higher values for sensitivity and specificity, with an AUC of 0.75 when the malignant breast cancer samples were compared with the control samples (Figure 3-A) and with an AUC of 0.72 when the benign patients were compared with the malignant patients (Figure 3-B). ROC curve analysis of miR-195 and let-7a did not show good sensitivity or specificity (data not shown).

## DISCUSSION

In the present study, we showed that the combination of the miR-195 and let-7a expression values presented good sensitivity and specificity in the sera of the control and benign groups compared with the serum of the malignant group. Even though these miRNAs have been described in a wide range of tumors, including breast cancer, the present study is innovative because it uses well-defined control groups, which exclude analysis bias. This study presents innovative biomarkers for the evaluation of breast cancer. Additionally, let-7a was found in exosomes, reinforcing the possibility of its detection in serum 27.

There is growing evidence that exosomal miRNAs that are secreted by donor cells are taken up by recipient or even cancer cells and that these miRNAs can be used as biomarkers 34. Exosomal miRNAs have pleiotropic roles in modulating many physiological and pathological processes, including cancer metastasis 35. There is evidence that tumor-derived exosomes facilitate tumor-stroma interactions and promote the formation of a supportive metastatic niche in distant organs, due in part to easier access to the vascular system 35.

Henegan et al. 24 studied the expression of circulating miRNAs in several tumor types (breast, colon, prostate, kidney, and melanoma) compared to that in normal individuals and found that miR-195 was upregulated only in breast cancer, suggesting that miR-195 could be a potential breast cancer biomarker, in association with let-7a. This study consisted of 83 women, and used a normal control group; however, the specific features of the control group were not described. The control groups were not described or well-defined and there was no description of the methods for the tissue analysis. This fact represents a bias, especially because both miRNAs are downregulated in a wide range of tumor types.

Interestingly, Sempere et al. 36 reported the downregulation of let-7a in breast cancer. Moreover, miR-195 was found to be downregulated in the circulation of a breast cancer animal model 26, in concordance with the present study. After a comparison of studies, discordance in the data, which can be explained by differences in the control group selection process and the number of patients who make up this group, becomes apparent. In the present study, a higher number of normal individuals, together with rigorous and well-established criteria, was used (Table 1).

In addition, reduced miR-195 expression in cancer progression has presented a correlation with poor overall survival in other cancer types, such as tongue squamous cell carcinoma 37 and multiform glioblastoma 38. Recently, Wang et al. 39 has described the role of miR-195 in metastasis, where reduced levels were associated with angiogenesis and worse recurrence-free survival in hepatocellular carcinoma cells. The authors suggest that miR-195 affects the expression of the proangiogenic factor *VEGF* and the pro-metastatic factors *VAV2* and *CDC42*. MiR-195 was negatively correlated with extracellular matrix (ECM) accumulation, reinforcing its role in metastasis 40. Recently, miR-195 was highlighted as a potentially predictive biomarker for the lymph node metastasis of gastric cancer 41.

The findings of the present study are consistent with a vast amount of evidence pointing to let-7a and miR-195 as tumor suppressors, whose downregulation and better sensitivity and specificity in combination provide evidence for their utilization as non-invasive biomarkers in breast cancer progression. Currently there are no good circulating biomarkers related to breast cancer. The known biomarkers, namely, CEA, CA15-3, and CA 27-29, present limited sensitivity and specificity and are not used for diagnostic or follow-up purposes in clinical practice 42. On the other hand, the combination of let-7a and miR-195 expression has been previously described as a potential circulating biomarker 25, and our findings indicate that this combination may be useful as a standard assessment, with an AUC of 0.72. Considering the absence of a better non-invasive biomarker in the literature and considering the lower costs of qPCR (approximately US$ 30.00), their efficacy should be considered, and further studies are necessary to confirm these findings.

## AUTHOR CONTRIBUTIONS

Marques MM conceived the study, provided advice during the study development and prepared the manuscript. Macedo T helped with realtime PCR experiments. Evangelista AF participated in the data collection and analysis and helped with the manuscript preparation. Vieira RA and Scapulatempo-Neto C provided clinical and pathology support in this study. Reis MR, Carvalho AL and Silva ID critically reviewed the manuscript. Silva ID also managed the financial support. All authors have reviewed and approved the final version of the manuscript.

## Figures and Tables

**Table 1:** Clinicopathological group characteristics.*

Group I - Healthy	Group II - Benign	Group III Neoplastic
n=72	n=56	n=72
Female	Female	Female
Asymptomatic	Appropriate clinical and radiological correlation	Appropriate clinical and radiological correlation
Normal mammography	Open biopsy	Open biopsy
Low cancer risk (Gail < 1.66)	Benign lesion at microscopy	Invasive ductal carcinoma
Absence of familial history	Lesion with absence of pathological cancer risk	Adequate TNM evaluation
		Unilateral tumor
		Absence of previous treatment
		Absence of second primary tumor

* Serum evaluation.

**Table 2:** Pathological features of breast cancer patients (Group 3).

Variable	Category	Number	%
TNM category(29)	I	14	19.4
	II	26	36.1
	III	26	36.1
	IV	6	8.3
TNM-T(29)	T1	23	31.9
	T2	26	36.1
	T3	11	15.3
	T4	12	16.7
TNM-N(29)	N0	29	40.3
	N1	24	33.3
	N2	8	11.1
	N3	11	15.3
Disease	Local	28	38.9
Extension*	Regional	38	52.8
	Metastasis	6	8.3
Estrogen	Positive	56	77.8
receptor	Negative	16	22.2
Progesterone	Positive	45	62.5
receptor	Negative	27	37.5
Her2	Positive	20	27.8
	Negative	52	72.2
Molecular	Luminal A/B Her2 -	44	61.1
Subtypes (30)	Luminal B Her2 +	13	18.1
	Triple negative	8	11.1
	Her 2	7	9.7
All	-	72	100.0

* Local=tumors N0, regional=tumors N+, metastasis=tumors M1.

## References

[1] Bartel DP (2004). MicroRNAs: genomics, biogenesis, mechanism, and function. Cell.

[2] Garzon R, Calin GA, Croce CM (2009). MicroRNAs in Cancer. Annu Rev Med.

[3] Almeida MI, Reis RM, Calin GA (2011). MicroRNA history: discovery, recent applications, and next frontiers. Mutat Res.

[4] Palmero EI, de Campos SG, Campos M, de Souza NC, Guerreiro ID, Carvalho AL (2011). Mechanisms and role of microRNA deregulation in cancer onset and progression. Genet Mol Biol.

[5] Calin GA, Sevignani C, Dumitru CD, Hyslop T, Noch E, Yendamuri S (2004). Human microRNA genes are frequently located at fragile sites and genomic regions involved in cancers. Proc Natl Acad Sci USA.

[6] Zhang B, Pan X, Cobb GP, Anderson TA (2007). microRNAs as oncogenes and tumor suppressors. Dev Biol.

[7] Roush S, Slack FJ (2008). The let-7 family of microRNAs. Trends Cell Biol.

[8] Finoux AL, Chartrand P (2008). [Oncogenic and tumour suppressor microRNAs]. Med Sci (Paris).

[9] Boominathan L (2010). The guardians of the genome (p53, TA-p73, and TA-p63) are regulators of tumor suppressor miRNAs network. Cancer Metastasis Rev.

[10] Frost RJ, Olson EN (2011). Control of glucose homeostasis and insulin sensitivity by the Let-7 family of microRNAs. Proc Natl Acad Sci USA.

[11] Kuehbacher A, Urbich C, Dimmeler S (2008). Targeting microRNA expression to regulate angiogenesis. Trends Pharmacol Sci.

[12] Huang Y (2012). A mirror of two faces: Lin28 as a master regulator of both miRNA and mRNA. Wiley Interdiscip Rev RNA.

[13] Dangi-Garimella S, Yun J, Eves EM, Newman M, Erkeland SJ, Hammond SM (2009). Raf kinase inhibitory protein suppresses a metastasis signalling cascade involving LIN28 and let-7. EMBO J.

[14] Chen PS, Su JL, Cha ST, Tarn WY, Wang MY, Hsu HC (2011). miR-107 promotes tumor progression by targeting the let-7 microRNA in mice and humans. J Clin Invest.

[15] Finnerty JR, Wang WX, Hébert SS, Wilfred BR, Mao G, Nelson PT (2010). The miR-15/107 group of microRNA genes: evolutionary biology, cellular functions, and roles in human diseases. J Mol Biol.

[16] Ding J, Huang S, Wang Y, Tian Q, Zha R, Shi H (2013). Genome-wide screening reveals that miR-195 targets the TNF-α/NF-κB pathway by down-regulating IκB kinase alpha and TAB3 in hepatocellular carcinoma. Hepatology.

[17] Flavin RJ, Smyth PC, Laios A, O’Toole SA, Barrett C, Finn SP (2009). Potentially important microRNA cluster on chromosome 17p13.1 in primary peritoneal carcinoma. Mod Pathol.

[18] Deng H, Guo Y, Song H, Xiao B, Sun W, Liu Z (2013). MicroRNA-195 and microRNA-378 mediate tumor growth suppression by epigenetical regulation in gastric cancer. Gene.

[19] Furuta M, Kozaki K, Tanimoto K, Tanaka S, Arii S, Shimamura T (2013). The tumor-suppressive miR-497-195 cluster targets multiple cell-cycle regulators in hepatocellular carcinoma. PLoS One.

[20] Xu T, Zhu Y, Xiong Y, Ge YY, Yun JP, Zhuang SM (2009). MicroRNA-195 suppresses tumorigenicity and regulates G1/S transition of human hepatocellular carcinoma cells. Hepatology.

[21] Bhattacharya A, Schmitz U, Wolkenhauer O, Schönherr M, Raatz Y, Kunz M (2013). Regulation of cell cycle checkpoint kinase WEE1 by miR-195 in malignant melanoma. Oncogene.

[22] Caramuta S, Lee L, Ozata DM, Akçakaya P, Xie H, Höög A (2013). Clinical and functional impact of TARBP2 over-expression in adrenocortical carcinoma. Endocr Relat Cancer.

[23] Li D, Zhao Y, Liu C, Chen X, Qi Y, Jiang Y (2011). Analysis of MiR-195 and MiR-497 expression, regulation and role in breast cancer. Clin Cancer Res.

[24] Heneghan HM, Miller N, Kelly R, Newell J, Kerin MJ (2010). Systemic miRNA-195 differentiates breast cancer from other malignancies and is a potential biomarker for detecting noninvasive and early stage disease. Oncologist.

[25] Heneghan HM, Miller N, Lowery AJ, Sweeney KJ, Newell J, Kerin MJ (2010). Circulating microRNAs as novel minimally invasive biomarkers for breast cancer. Ann Surg.

[26] Waters PS, McDermott AM, Wall D, Heneghan HM, Miller N, Newell J (2012). Relationship between circulating and tissue microRNAs in a murine model of breast cancer. PLoS One.

[27] Kharaziha P, Ceder S, Li Q, Panaretakis T (2012). Tumor cell-derived exosomes: a message in a bottle. Biochim Biophys Acta.

[28] Vogel VG (2009). The NSABP Study of Tamoxifen and Raloxifene (STAR) trial. Expert Rev Anticancer Ther.

[29] Edge SB, Byrd DR, Compton CC, Fritz AG, Greene FL, Trotti A (2010). Breast. AJCC Cancer Staging Manual.

[30] Goldhirsch A, Wood WC, Coates AS, Gelber RD, Thurlimann B, Senn HJ (2011). Strategies for subtypes dealing with the diversity of breast cancer: highlights of the St. Gallen international expert consensus on the primary therapy of early breast Cancer. Ann. Oncol.

[31] (2017). R: The R Project for Statistical Computing.

[32] Pfaffl MW (2001). A new mathematical model for relative quantification in real-time RT-PCR. Nucleic Acids Res.

[33] (2017). ROCR: Visualizing the Performance of Scoring Classifiers.

[34] Bullock MD, Silva AM, Kanlikilicer-Unaldi P, Filant J, Rashed MH, Sood AK (2015). Exosomal Non-Coding RNAs: Diagnostic, Prognostic and Therapeutic Applications in Cancer. Non-Coding RNA.

[35] Alečković M, Kang Y (2015). Regulation of cancer metastasis by cell-free miRNAs. Biochim Biophys Acta.

[36] Sempere LF, Christensen M, Silahtaroglu A, Bak M, Heath CV, Schwartz G (2007). Altered MicroRNA expression confined to specific epithelial cell subpopulations in breast cancer. Cancer Res.

[37] Jia LF, Wei SB, Gong K, Gan YH, Yu GY (2013). Prognostic implications of MicoRNA miR-195 expression in human tongue squamous cell carcinoma. PLoS One.

[38] Lakomy R, Sana J, Hankeova S, Fadrus P, Kren L, Lzicarova E (2011). MiR-195, miR-196b, miR-181c, miR-21 expression levels and O-6-methylguanine-DNA methyltransferase methylation status are associated with clinical outcome in glioblastoma patients. Cancer Sci.

[39] Wang R, Zhao N, Li S, Fang JH, Chen MX, Yang J (2013). MicroRNA-195 suppresses angiogenesis and metastasis of hepatocellular carcinoma by inhibiting the expression of VEGF, VAV2 and CDC42. Hepatology.

[40] Chen H, Untiveros GM, McKee LA, Perez J, Li J, Antin PB (2012). Micro-RNA-195 and -451 regulate the LKB1/AMPK signaling axis by targeting MO25. PLoS ONE.

[41] Wu WY, Xue XY, Chen ZJ, Han SL, Huang YP, Zhang LF (2011). Potentially predictive microRNAs of gastric cancer with metastasis to lymph node. World J Gastroenterol.

[42] Sturgeon C (2002). Practice guidelines for tumor marker use in the clinic. Clin Chem.

